# Prevalence and clinical markers of herpes simplex virus infection in oral lesions of bullous pemphigoid

**DOI:** 10.3389/fimmu.2024.1387503

**Published:** 2024-04-18

**Authors:** Hanmei Zhang, Meiwen Yu, Guirong Liang, Suo Li, Chenjing Zhao, Ke Jing, Suying Feng

**Affiliations:** Hospital for Skin Diseases, Institute of Dermatology, Chinese Academy of Medical Sciences & Peking Union Medical College, Nanjing, China

**Keywords:** Bullous pemphigoid, autoimmune subepidermal bullous disease, oral lesions, HSV, infection, prevalence, clinical markers

## Abstract

**Background:**

The manifestations of bullous pemphigoid (BP) and herpes simplex virus (HSV) infection are similar in oral mucosa, and the laboratory detection of HSV has some limitations, making it difficult to identify the HSV infection in oral lesions of BP. In addition, the treatments for BP and HSV infection have contradictory aspects. Thus, it is important to identify the HSV infection in BP patients in time.

**Objective:**

To identify the prevalence and clinical markers of HSV infection in oral lesions of BP.

**Methods:**

This prospective cross-sectional descriptive analytical study was conducted on 42 BP patients with oral lesions. A total of 32 BP patients without oral lesions and 41 healthy individuals were enrolled as control groups. Polymerase chain reaction was used to detect HSV. Clinical and laboratory characteristics of patients with HSV infection were compared with those without infection.

**Results:**

A total of 19 (45.2%) BP patients with oral lesions, none (0.0%) BP patients without oral lesions, and four (9.8%) healthy individuals were positive for HSV on oral mucosa. Among BP patients with oral lesions, the inconsistent activity between oral and skin lesions (p=0.001), absence of blister/blood blister in oral lesions (p=0.020), and pain for oral lesions (p=0.014) were more often seen in HSV-positive than HSV-negative BP patients; the dosage of glucocorticoid (p=0.023) and the accumulated glucocorticoid dosage in the last 2 weeks (2-week AGC dosage) (p=0.018) were higher in HSV-positive BP patients. Combining the above five variables as test variable, the AUC was 0.898 (p<0.001) with HSV infection as state variable in ROC analysis. The absence of blister/blood blister in oral lesions (p=0.030) and pain for oral lesions (p=0.038) were found to be independent predictors of HSV infection in multivariable analysis. A total of 14 (73.7%) HSV-positive BP patients were treated with 2-week famciclovir and the oral mucosa BPDAI scores significantly decreased (p<0.001).

**Conclusion:**

HSV infection is common in BP oral lesions. The inconsistent activity between oral and skin lesions, absence of blister in oral lesions, pain for oral lesions, higher currently used glucocorticoid dosage, and higher 2-week AGC dosage in BP patients should alert physicians to HSV infection in oral lesions and treat them with 2-week famciclovir in time.

## 1 Introduction

Bullous pemphigoid (BP) is an autoimmune subepidermal bullous dermatosis induced by autoantibodies against BP180 and BP230, the important components of the dermal–epidermal junction. It mainly manifests as tense blisters, urticarial plaques, erythema, and erosions on the skin with severe pruritus. Approximately 10%–20% of BP patients are accompanied by erosions or blisters on the mucosa, mainly on the oral mucosa (1–4).

Herpes simplex virus (HSV) has two subtypes, HSV-1 and HSV-2. HSV spreads widely among people, with 52%–91% of people reportedly HSV-1 seropositive and 4%–24% of people reportedly HSV-2 seropositive (5–8). The clinical manifestations of HSV infection are diverse, which include eczema herpetiform, herpetic gingivostomatitis, herpes labialis, genital herpes, and asymptomatic infection. HSV-1 mainly causes mucocutaneous infection outside the genitals, especially around the mouth or nose, and HSV-2 mainly causes mucocutaneous infection around the genital area (9).

On the skin, BP typically manifests as diffused urticarial plaques and blisters all around the body with severe pruritus, while HSV infection often appears as clustered vesicles and erosions around the mouth, nose, or vulva with pain, burning, pruritus, or tingling, which can distinguish the HSV infection from BP. However, on the oral mucosa, both BP and HSV infection present with erosions and blisters (1, 10). The similarity in manifestations between BP and HSV infection on oral mucosa makes it difficult to identify the HSV infection in oral lesions of BP patients.

The laboratory detection of HSV presents several limitations. HSV-specific IgG antibody tests have low specificity due to the high prevalence of HSV among people. Although a significant rise in the HSV-specific IgG antibody titer can provide diagnostic evidence for active HSV infection, it is less practical in clinical work because of the need for frequent follow-up visits. HSV-specific IgM antibody tests suffer from low sensitivity because of the short duration of IgM antibodies. Other methods, including viral culture, cytological smear, direct fluorescent antibody studies, tissue biopsy, and viral deoxyribonucleic acids (DNA) detection by polymerase chain reaction (PCR), are all challenging to practice and promote due to the high requirements for personnel, testing environment, and equipment (9). The limitations of HSV detection in clinical practice exacerbate the difficulty of identifying the HSV infection in oral lesions of BP patients in time.

The treatments for HSV infection and BP have some contradictions. HSV infection is mainly treated with antivirus drugs such as acyclovir, valacyclovir, and famciclovir. Maintaining an immunocompetent status is beneficial for suppressing HSV. Conversely, BP as an autoimmune disease is mainly treated by glucocorticoid or other immunosuppressants, which can, however, make BP patients immunocompromised and trigger HSV activation, prolong HSV shedding, and increase the risk of HSV dissemination (11). Hence, it is important to identify the HSV infection in BP patients in time.

Nikkels et al. found that there were 10% (2/20) of skin biopsies from BP patients positive for HSV by immunohistochemistry and PCR (12), while studies that explore the prevalence or clinical markers of HSV infection in oral lesions of BP are still absent.

In this study, we investigated the prevalence and clinical markers of HSV infection in oral lesions of BP patients to find out the importance of HSV infection among oral lesions of BP and provide some clinical clues for clinicians to identify HSV infection in time in oral lesions of BP, which could avoid the unnecessary adjustment of glucocorticoid or other immunosuppressants and add the antiviral treatment in time.

## 2 Materials and methods

### 2.1 Study design

We conducted a prospective cross-sectional descriptive analytical study on BP patients with oral lesions who were admitted to our hospital from 1 January 2022 to 30 June 2023. BP patients without oral lesions and healthy individuals were enrolled as controls during the study. The Ethics Committee of the Chinese Academy of Medical Sciences, Hospital for Skin Diseases (2021-KY-060) approved the project. Written informed consents were obtained from all participants after a thorough explanation of study procedures and objectives.

### 2.2 Inclusion and exclusion criteria

Patients who were diagnosed with BP by clinical manifestation, biopsy, direct immunofluorescence, enzyme-linked immunosorbent assay or immunoblot, and indirect immunofluorescence using salt-split skin (13), and accompanied by oral lesions, regardless of the treatment status and the disease activity, were included in the study.

Patients were excluded from the study if they had met at least one of the following criteria: patients who could not exclude the diagnosis of mucous membrane pemphigoid; or patients who were accompanied by another autoimmune bullous disease, such as P200 pemphigoid, epidermolysis bullosa acquisita; or patients who were accompanied by other diseases that might affect the oral mucosa, such as Behet’s disease, oral lichen planus, Stevens–Johnson syndrome, and oral cancer.

### 2.3 Clinical assessment and laboratory examination of BP

Clinical examinations of the skin and mucosa were performed carefully for each patient to assess the location, basement, type, and shape of the lesions, and the disease severity was assessed by bullous pemphigoid disease area index (BPDAI) (14).

The severity of oral lesions’ pain was assessed on a 3-grade scale: (i) mild, discomfort or pain with firm pressure that does not hinder eating or speaking; (ii) moderate, pain with gentle pressure that affects eating but not speaking or drinking; and (iii) severe, persistent pain without any pressure, which impedes speaking or drinking.

The disease activity of BP was divided into active and inactive. If the new lesions or pruritic symptoms cease to form and the established lesions begin to heal or even have disappeared, the disease will be judged as inactive; if not, the disease will be judged as active. In this study, we assessed the disease activity separately for oral and skin lesions. For BP patients, whose oral lesions were active but the skin lesions were inactive or whose oral lesions were inactive but the skin lesions were active, the disease activity would be defined as an inconsistent activity between oral and skin lesions.

Other clinical information of the patients, which included age, sex, comorbidities, duration of the skin and oral lesions up to the time of swabs taking, dosage and duration of the glucocorticoid usage when the swabs were taken, accumulated glucocorticoid dosage in the last 2 weeks (2-week AGC dosage), and other treatment information, was also collected and recorded.

Laboratory tests such as the titers of anti-BP180 NC16A antibody and anti-BP230 antibody, and the counts and percentages of immune cells, and the amount of albumin, globulin, total protein, and immunoglobulins in serum samples, were performed and collected when the patients were enrolled.

### 2.4 Sample collection and HSV detection

HSV-1 and HSV-2 were detected from the oral swabs of participants by PCR. For BP patients with oral lesions, swabs were taken from the oral lesions; for BP patients without oral lesions and healthy individuals, swabs were taken from the entire oral mucosa.

**Figure 1:**
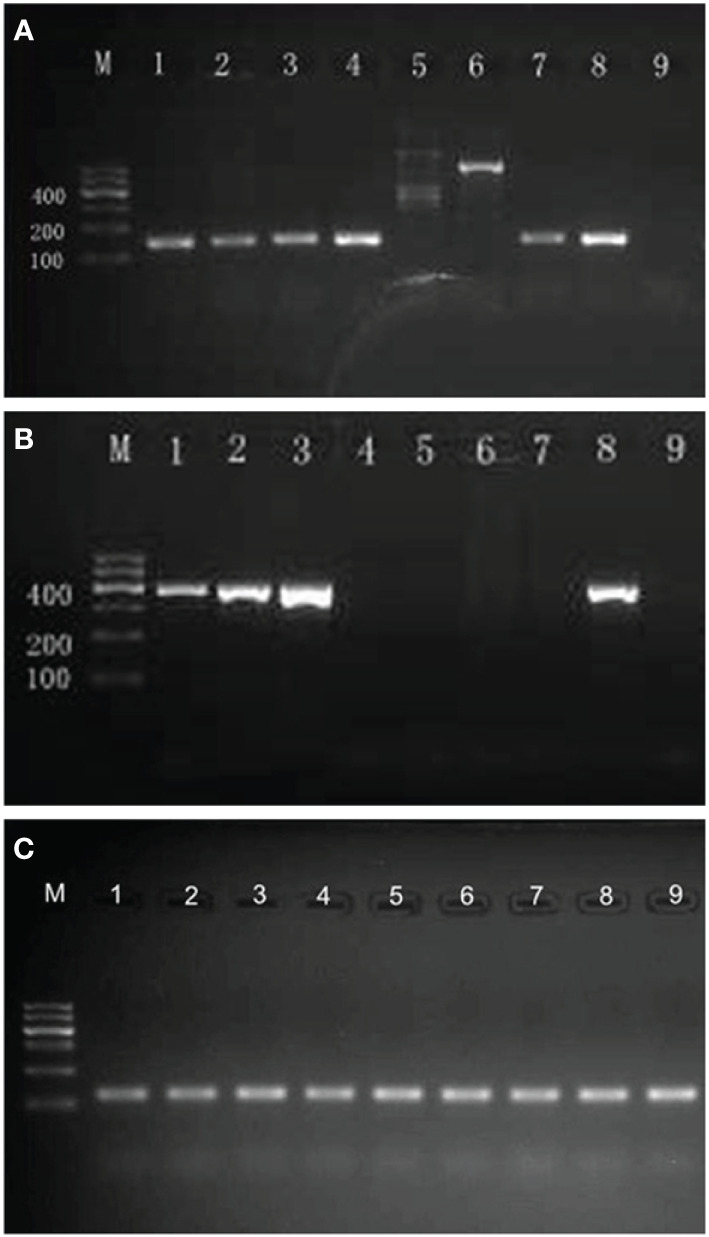
Results of agarose gel electrophoresis of PCR products. **(A)** Agarose gel electrophoresis of PCR products for HSV-1 DNA (142 bp). M, molecular weight marker (DNA ladder, 100 bp); lane 1–4,7, positive for HSV-1; lane 5,6, negative for HSV-1; lane 8, positive control for HSV-1; lane 9, negative control for HSV-1. **(B)** Agarose gel electrophoresis of PCR products for HSV-2 DNA (391 bp). M, molecular weight marker (DNA ladder, 100 bp); lanes 1–3, positive for HSV-2; lane 4–7, negative for HSV-2; lane 8, positive control for HSV-2; lane 9, negative control for HSV-2. **(C)** Agarose gel electrophoresis of PCR products for β2-microglobulin DNA (102 bp). M, molecular weight marker (DNA ladder, 100 bp); all samples were detected positive for β2-microglobulin DNA.

DNA was extracted from the swabs using DNA extraction buffer (Da An Gene, China) according to the manufacturer’s instructions. Five microliters of the extracted DNA was used as template in a final volume of 50 μl PCR mixture containing 25 μl of 2× GoTaq^®^ Green Master Mix (Promega), 16 μl of nuclease-free water (Promega), and 2 μl of upstream and downstream primers (10 μmol/l, Sangon Biotech, China) for HSV-1, HSV-2, or β2-microglobulin. The sequences of the oligonucleotide primers used in PCR for HSV-1, HSV-2, and β2-microglobulin DNA detection are shown in **Table 1** (15–17). Nuclease-free water (Promega) and HSV-1/HSV-2 positive DNA (provided by the sexually transmitted disease laboratory) were taken separately as negative and positive controls. The reaction was carried out in a DNA thermal cycler (GeneAmp^®^ PCR System 9700, ABI, USA). Temperature cycling was as follows: 95°C for 5 min; 95°C over 45 s, 57°C over 45 s (HSV-1/HSV-2)/50°C over 45 s (β2-microglobulin), and 72°C over 1 min for 35 cycles; and 72°C for 5 min. The amplification products were separated by 2% agarose gel electrophoresis and visualized by subsequent 4S Red Plus Nucleic Acid Stain (Sangon Biotech, China) staining and UV radiation. In positive cases, a distinct and sharp band of the expected size was seen (**Figure 1**), and the results were verified by Sanger sequencing for the PCR product (Sangon Biotech, China).

**Table 1:** Sequences of the oligonucleotide primers used in PCR for HSV-1, HSV-2, and β2-microglobulin DNA detection.

Primers	Direction	Sequences	Size
HSV-1 (15)	for	5′-CCCTGTCTCGCGCGACCGAC-3’	142 bp
	rev	5′-TCACCGACCCATACGCGTAA-3’	
HSV-2 (16)	for	5′-ATGGTGAACATCGACATGTACGG-3’	391 bp
	rev	5′-CCTCCTTGTCGAGGCCCCGAAAC-3’	
β2-microglobulin (17)	for	5′-TCCAACATCAACATCTTGGT-3’	102 bp
	rev	5′-TCCCCCAAATTCTAAGCAGA-3’	

Participants who were detected positive for HSV were informed of the results by phone. The HSV-positive BP patients with oral lesions were treated with oral famciclovir 0.25g, three times daily for 2 weeks, with the dosage of glucocorticoid decreased or maintaining the existing treatments. The disease activity and BPDAI scores of oral lesions were assessed again by phone or clinic visit after the 2-week famciclovir treatment. The oral lesions were retested for HSV by PCR after 1 and 2 weeks of famciclovir treatment if the oral swabs were available. The HSV-positive participants without oral lesions were followed up by phone for oral lesions development 1 and 2 weeks after the oral swabs were taken.

### 2.5 Statistical analysis

Statistical analysis was performed using IBM SPSS 26.0 software. Descriptive statistics were used for categorical variables. Mean with standard deviation (SD) or median with interquartile range (IQR) was estimated for continuous variables. The chi-square test/Fisher’s exact test was used to compare the differences in categorical variables. The one-way ANOVA analysis, independent t-test, paired t-test, or Mann–Whitney U-test was used to compare the differences in continuous variables. Variables in the univariate analysis with a p<0.05 were taken up for multivariate analysis by binary regression analysis to identify the independent parameters of HSV infection. ROC analysis was conducted to figure out the clinical markers of HSV infection in oral lesions of BP. Results were considered statistically significant when the two-sided p<0.05.

## 3 Results

### 3.1 Study population

In this study, we enrolled 42 BP patients with oral lesions as the lesion group, 32 BP patients without oral lesions as the non-lesion group, and 41 healthy individuals as the healthy control group.

The mean age (SD) of the participants was 67.4 (11.8) years in the lesion group, 65.6 (17.1) years in the non-lesion group, and 62.2 (9.8) years in the healthy control group. The male-to-female ratio of the three groups was 1.5:1, 2.2:1, and 1.2:1, respectively. Neither age (p=0.174) nor sex distribution (p=0.425) was statistically different among the three groups. Among all the participants, except for two patients in the lesion group accompanied by herpes labialis, all individuals did not have any suggestive symptoms of HSV infection.

In lesion and non-lesion groups, hypertension was the most common comorbidity, followed by diabetes. Glucocorticoid was the most commonly used drug, followed by immunosuppressants and tetracyclines. The comorbidity, treatment, and disease activity of skin lesions did not show significant differences between the lesion and non-lesion groups (**Table 2**).

**Table 2:** Characteristics of bullous pemphigoid patients in lesion and non-lesion groups.

Parameters	Lesion group (n=42)	Non-lesion group (n=32)	p-value
**Age, y, mean (SD)**	67.4 (11.8)	65.6 (17.1)	0.591^a^
**Sex, n (%)**			0.414^b^
Female	17 (40.5)	10 (31.3)	
Male	25 (59.5)	22 (68.7)	
**Comorbidity, n (%)**	23 (54.8)	18 (56.3)	0.898^b^
Hypertension	14 (33.3)	11 (34.4)	
Diabetes	7 (16.7)	7 (21.9)	
Malignant tumor	3 (7.1)	2 (6.3)	
Psoriasis	3 (7.1)	1 (3.1)	
Others^c^	11 (26.2)	2 (6.3)	
Treatment drugs, n (%)			
None	11 (26.2)	7 (21.9)	0.668^b^
GC alone	12 (28.6)	10 (31.3)	0.803^b^
GC with others	15 (35.7)	11 (34.4)	0.905^b^
GC with tetracyclines	5 (11.9)	4 (12.5)	
GC with immunosuppressants	8 (19.0)	5 (15.6)	
GC with baricitinib	0 (0.0)	2 (6.3)	
GC with dupilumab	4 (9.5)	2 (6.3)	
GC with IVIG	2 (4.8)	0 (0.0)	
Tetracyclines alone	2 (4.8)	2 (6.3)	>0.99^d^
Baricitinib alone	1 (2.4)	0 (0.0)	>0.99^d^
Dupilumab alone	1 (2.4)	0 (0.0)	>0.99^d^
Immunosuppressants alone	0 (0.0)	1 (3.1)	0.432^d^
Immunosuppressants with dupilumab	0 (0.0)	1 (3.1)	0.432^d^
**Treatment duration, m, median (IQR)**	0.5 (0.1-2.0)	1.3 (0.1-5.3)	0.092^e^
**Disease activity of skin lesions, n (%)**			0.316^b^
Active	22 (52.4)	13 (40.6)	
Inactive	20 (47.6)	19 (59.4)	

y, years; SD, standard deviation; GC, glucocorticoid; IVIG, intravenous immunoglobin; IQR, interquartile range; m, months.

a p-value based on independent’s t-test.

b p-value based on person χ^2^ statistic.

c Other comorbidities included hepatitis B, asthma, pulmonary embolism, renal insufficiency, cerebral infarction, Parkinson’s disease, rheumatoid arthritis, Sjogren’s syndrome, atrial fibrillation, and coronary heart disease.

d p-value based on Fisher’s exact test.

e p-value based on Mann–Whitney U-test.

Among the 42 BP patients in the lesion group, 22 (52.4%) patients were active in both oral lesions and skin lesions, three (7.1%) patients were inactive in both oral lesions and skin lesions, and 17 (40.5%) patients were active in oral lesions but inactive in skin lesions. A total of 30 (71.4%) patients developed skin lesions earlier than oral lesions, 11 (26.2%) patients developed skin lesions and oral lesions at the same time, and only one (2.4%) patient developed skin lesions later than oral lesions. The oral lesions of BP were mostly located on the buccal mucosa and then followed by the palate/posterior pharynx and gingiva. The median (IQR) oral mucosa BPDAI score of the enrolled BP patients in the lesion group was 5.0 (2.0–8.1), and about half of them were painful for oral lesions, but the pain was mostly mild (**Table 3**). Additionally, there were also four patients with lesions on extra-oral mucosa, including two patients with lesions on genital mucosa and two on nasal mucosa.

**Table 3:** Clinical characteristics of patients with HSV infection compared with those without HSV infection in the lesion group.

Characteristics	All cases (n=42)	HSV^+^ patients (n=19)	HSV^−^ patients (n=23)	p-value
**Age, y, mean (SD)**	67.4 (11.8)	65.2 (9.1)	69.2 (13.5)	0.277 ^a^
**Sex, n (%)**				0.408^b^
Female	17 (40.5)	9 (47.4)	8 (34.8)	
Male	25 (59.5)	10 (52.6)	15 (65.2)	
**Disease activity of oral and skin lesions, n (%)**				**0.001** ^b^
Consistent	25 (59.5)	6 (31.6)	19 (82.6)	
Inconsistent	17 (40.5)	13 (68.4)	4 (17.4)	
**Oral lesions later than skin lesions, n (%)**	30 (71.4)	13 (68.4)	17 (73.9)	0.742^b^
**Perioral or perinasal erosions/blisters, n (%)**	10 (23.8)	5 (26.3)	5 (21.7)	>0.99 ^c^
Signs and symptoms of oral lesions, n (%)				
Location				
Buccal mucosa	29 (69.0)	14 (73.7)	15 (65.2)	0.555^b^
Palate/posterior pharynx	22 (52.4)	8 (42.1)	14 (60.9)	0.226 ^b^
Gingiva	18 (42.9)	9 (47.4)	9 (39.1)	0.591 ^b^
Tongue/floor of mouth	10 (23.8)	5 (26.3)	5 (21.7)	>0.99 ^c^
Labial mucosa	14 (33.3)	6 (31.6)	8 (34.8)	0.826 ^b^
Lesion type				
Blister/blood blister	17 (40.5)	4 (21.1)	13 (56.5)	**0.020** ^b^
Erythema	21 (50.0)	10 (52.6)	11 (47.8)	0.757 ^b^
Erosion	34 (81.0)	16 (84.2)	18 (78.3)	0.709 ^c^
Shape				
Suborbicular	37 (88.1)	16 (84.2)	21 (91.3)	0.644^c^
Irregular	12 (28.6)	7 (36.8)	5 (21.7)	0.281^b^
Cord-like/linear	3 (7.1)	1 (5.3)	2 (8.7)	>0.99 ^c^
Yellowish-white pseudomembrane	28 (66.7)	15 (78.9)	13 (56.5)	0.125^b^
Pain	20 (47.6)	13 (68.4)	7 (30.4)	**0.014** ^b^
mild	12 (28.6)	9 (47.4)	3 (13.0)	**0.014** ^b^
moderate	6 (14.3)	2 (10.5)	4 (17.4)	0.673 ^c^
severe	2 (4.8)	2 (10.5)	0 (0.0)	0.199^c^
**Duration of oral lesions, d, median (IQR)**	10.0 (3.0–30.0)	14.0 (3.0–30.0)	7.0 (3.0–30.0)	0.621^d^
Disease severity, median (IQR)				
Oral mucosa BPDAI	5.0 (2.0–8.1)	5.0 (2.0–7.0)	4.0 (1.0–10.3)	0.559 ^d^
Skin BPDAI	27.0 (8.9–54.0)	28.3 (15.6–48.0)	26.3 (3.6–63.0)	0.939 ^d^
Total BPDAI	35.0 (10.2–61.0)	35.0 (19.9–54.0)	31.8 (5.6–67.3)	0.870 ^d^
Treatment				
None, n (%)	11 (26.2)	2 (10.5)	9 (39.1)	0.075 ^c^
GC, n (%)	27 (64.3)	15 (78.9)	12 (52.2)	0.071^b^
Dosage of GC^e^, mg/d, median (IQR)	22.5 (0.0–67.5)	40.0 (10.0–75.0)	5.0 (0.0–30.0)	**0.023** ^d^
Duration of GC usage, m, median (IQR)	0.3 (0.0–1.1)	0.5 (0.1–0.8)	0.1 (0.0–2.0)	0.229^d^
Two-week AGC^e^, mg, median (IQR)	175.0 (0.0–551.0)	285.0 (112.5–1050.0)	70.0 (0.0–410.0)	**0.018** ^d^
Tetracyclines, n (%)	7 (16.7)	4 (21.1)	3 (13.0)	0.682^c^
Baricitinib, n (%)	1 (2.4)	1 (5.3)	0 (0.0)	0.452^c^
Dupilumab, n (%)	5 (11.9)	4 (21.1)	1 (4.3)	0.158^c^
Immunosuppressants, n (%)	8 (19.0)	5 (26.3)	3 (13.0)	0.433^c^
**Duration of treatment, m, median (IQR)**	0.5 (0.0–1.6)	0.6 (0.3–1.5)	0.2 (0.0–2.0)	0.261^d^

Bold p-value indicates p < 0.05.

y, years; SD, standard deviation; d, days; IQR, interquartile range; BPDAI, Bullous Pemphigoid Disease Area Index; GC, glucocorticoid; Two-week AGC, the accumulated glucocorticoid dosage in the last 2 weeks; m, months.

a p-value based on independent’s t-test.

b p-value based on person χ^2^ statistic.

c p-value based on Fisher’s exact test.

d p-value based on Mann–Whitney U-test.

e The dosage of glucocorticoid was all converted to prednisone equivalent.

### 3.2 Results of the HSV detection

In the lesion group, HSV was positive in 19 (45.2%) cases detected by PCR, among whom 18 (42.9%) were positive for HSV-1 and one (2.4%) was positive for HSV-2. In the non-lesion group, none of the patients was positive for HSV. In the healthy control group, HSV was positive in four (9.8%) individuals, and all of them were positive for only HSV-1. For the positive rate of HSV, the lesion group was significantly higher than the non-lesion and healthy control groups (both p<0.001), while the latter two groups showed no statistical difference (p=0.126).

### 3.3 Comparison of clinical and laboratory characteristics between BP patients with and without HSV infection in oral lesions

BP patients in the lesion group were divided into HSV-positive (19 cases) and HSV-negative (23 cases) subgroups according to the results of HSV detection. The clinical characteristics of the two subgroups were compared in many aspects, and the results are shown in **Table 3**. Compared to the HSV-negative subgroup, the inconsistent activity between oral and skin lesions (p=0.001), absence of blister/blood blister in oral lesions (p=0.020), and pain for oral lesions (p=0.014) were more often seen in the HSV-positive subgroup; the dosage of glucocorticoid (p=0.023) and 2-week AGC dosage (p=0.018) were higher in the HSV-positive subgroup. The age, sex distribution, development order of oral and skin lesions; proportion of perioral or perinasal lesions appearing; disease severity; duration of treatment and glucocorticoid usage; and location, shape, basement, and duration of oral lesions showed no statistical differences between the two subgroups (**Table 3**).

In addition to the clinical characteristics, the laboratory tests were also compared between HSV-positive and HSV-negative subgroups (**Table 4**). However, all the parameters that include the titer of BP180 autoantibody, positive rate, and titer of BP230 autoantibody, elevated rate of the count and percentage of immune cells in blood routine test, and the amount of total protein, albumin, globulin, and immunoglobulins in serum samples were not statistically different between the two subgroups (**Table 4**).

**Table 4:** Laboratory tests of patients with HSV infection compared with those without HSV infection in the lesion group.

Laboratory tests	All cases(n=42)	HSV^+^ patients (n=19)	HSV^−^ patients (n=23)	p-value
BP180 autoantibody^a^, U/ml, median (IQR)	89.0 (41.3–145.7)	124.0 (43.0–467.0)	66.3 (39.1–127.3)	0.120^b^
Positive of BP230 autoantibody^c^, n (%)	15 (35.7)	5 (26.3)	10 (43.5)	0.248^d^
BP230 autoantibody ^e^, U/ml, median (IQR)	40.7 (25.7–61.8)	55.7 (28.6–230.7)	36.5 (25.4–70.4)	0.513^b^
Blood routine test^f^, n (%)				
Elevated WBC count	16 (41.0)	5 (29.4)	11 (50.0)	0.195^d^
Elevated NE count	17 (43.6)	6 (35.3)	11 (50.0)	0.358^d^
Elevated NE percentage	12 (30.8)	5 (29.4)	7 (31.8)	0.872^d^
Elevated LC count	2 (5.1)	0 (0.0)	2 (9.1)	0.495^g^
Elevated LC percentage	1 (2.6)	0 (0.0)	1 (4.5)	>0.999^g^
Elevated EO count	13 (33.3)	5 (29.4)	8 (36.4)	0.648^d^
Elevated EO percentage	10 (25.6)	4 (23.5)	6 (27.3)	>0.999^g^
Elevated MO count	17 (43.6)	10 (58.8)	7 (31.8)	0.092^d^
Elevated MO percentage	6 (15.4)	5 (29.4)	1 (4.5)	0.068^g^
Serum proteins^h^, g/L, mean (SD)				
Total protein	63.5 (9.1)	63.0 (10.2)	63.9 (8.0)	0.800^i^
Albumin	35.3 (5.5)	35.2 (6.1)	35.5 (5.1)	0.862^i^
Globulin	28.2 (5.6)	27.9 (6.5)	28.4 (4.8)	0.812^i^
Immunoglobulins				
IgG^j^, g/L, mean (SD)	11.6 (4.0)	10.9 (3.8)	12.6 (4.3)	0.446^i^
IgA^j^, g/L, mean (SD)	2.3 (1.0)	2.2 (1.2)	2.4 (0.6)	0.823^i^
IgM^j^, g/L, median (IQR)	0.6 (0.4–0.9)	0.6 (0.4–0.8)	0.6 (0.3–1.3)	0.864^b^
IgE^k^, g/L, median (IQR)	467.0 (188.0–1977.8)	367.0 (146.0–1068.3)	789.5 (189.8–2369.8)	0.334^b^

IQR, interquartile range; WBC, white blood cell; NE, neutrophil; LC, lymphocyte; EO, eosinophils; MO, monocyte; SD, standard deviation; Ig, immunoglobulin.

a The titer of BP180 autoantibody was detected with the conventional BP180NC16A ELISA kits (MBL, Nagoya, Japan) following the manufacturer’s instructions. The threshold value was 9 U/ml.

b p-value based on Mann–Whitney U-test.

c The titer of BP230 autoantibody was detected with the BP230 ELISA kits (Euroimmun, Lübeck, Germany) following the manufacturer’s instructions. The threshold value was 20 U/ml.

d p-value based on Pearson χ^2^ statistic.

e The titer of BP230 autoantibody calculated in this table only includes patients whose BP230 autoantibody was detected positive.

f There were 39 patients in total in the lesion group detected blood routine test, among whom 17 were HSV-positive patients and 22 were HSV-negative patients.

g p-value based on Fisher’s exact test.

h There were 33 patients in total in the lesion group detected serum proteins, among whom 17 were HSV-positive patients and 16 were HSV-negative patients.

i p-value based on independent’s t-test.

j There were 15 patients in total in the lesion group detected IgG, IgA, and IgM, among whom nine were HSV-positive patients and six were HSV-negative patients.

k There were 24 patients in total in lesion group detected IgE, among whom 10 were HSV-positive patients and 14 were HSV-negative patients.

Furthermore, a multivariable model was established to identify the independent predictors of HSV infection in oral lesions of BP. In this model, HSV infection was set as the dependent variable and inconsistent activity between oral and skin lesions, absence of blister/blood blister in oral lesions, pain for oral lesions, dosage of glucocorticoid, and 2-week AGC dosage were set as independent variables. After adjustment, the absence of blister/blood blister in oral lesions (p=0.030) and pain for oral lesions (p=0.038) were found to be independently associated with HSV infection.

### 3.4 Clinical markers of HSV infection in oral lesions of BP patients

**Figure 2:**
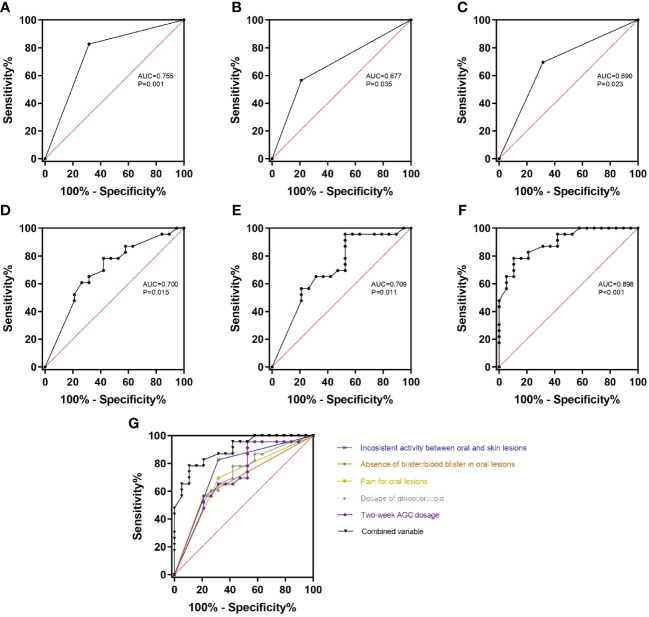
ROC curves of the variables with significant differences between HSV-positive and HSV-negative subgroups. **(A–E)** The inconsistent activity between oral and skin lesions **(A)**, absence of blister/blood blister in oral lesions **(B)**, pain for oral lesions **(C)**, dosage of glucocorticoid **(D)**, and 2-week AGC dosage **(E)** can significantly distinguish HSV-positive subgroup from HSV-negative subgroup (all p<0.05). **(F)** All the above variables were combined as the test variable, and the combined variable can get the highest diagnosis probability (AUC=0.898, p<0.001). **(G)** All the ROC curves were organized together to directly show that the combined variable has the biggest AUC. AUC, area under the curve; 2-week AGC dosage, accumulated glucocorticoid dosage in the last 2 weeks.

According to the above results, there were indeed some differences between BP patients with and without HSV infection in oral mucosa. To figure out whether these variables could serve as clinical markers to help indicate the HSV infection in oral lesions of BP, ROC analysis was done to calculate the prediction probability of the variables involved in the present study. First, the variables were analyzed individually for the area under the curve (AUC). The results showed that the inconsistent activity between oral and skin lesions (AUC=0.755, p=0.001), absence of blister/blood blister in oral lesions (AUC=0.677, p=0.035), pain for oral lesions (AUC=0.690, p=0.023), dosage of glucocorticoid (AUC=0.700, p=0.015, cut-off value=33.75mg/d prednisone equivalent with a sensitivity of 0.579 and specificity of 0.783), and 2-week AGC dosage (AUC=0.709, p=0.011, cut-off value=554mg prednisone equivalent with a sensitivity of 0.474 and specificity of 0.957) could significantly distinguish oral lesions of BP with HSV infection from those without HSV infection (**Figures 2A–E**). Then, all the aforementioned variables were combined into a single test variable to calculate AUC, resulting in an improved AUC of 0.898 with the highest Youden index of 0.678 (p<0.001) (**Figures 2F, G**).

### 3.5 Antiviral therapy for BP patients

**Figure 3:**
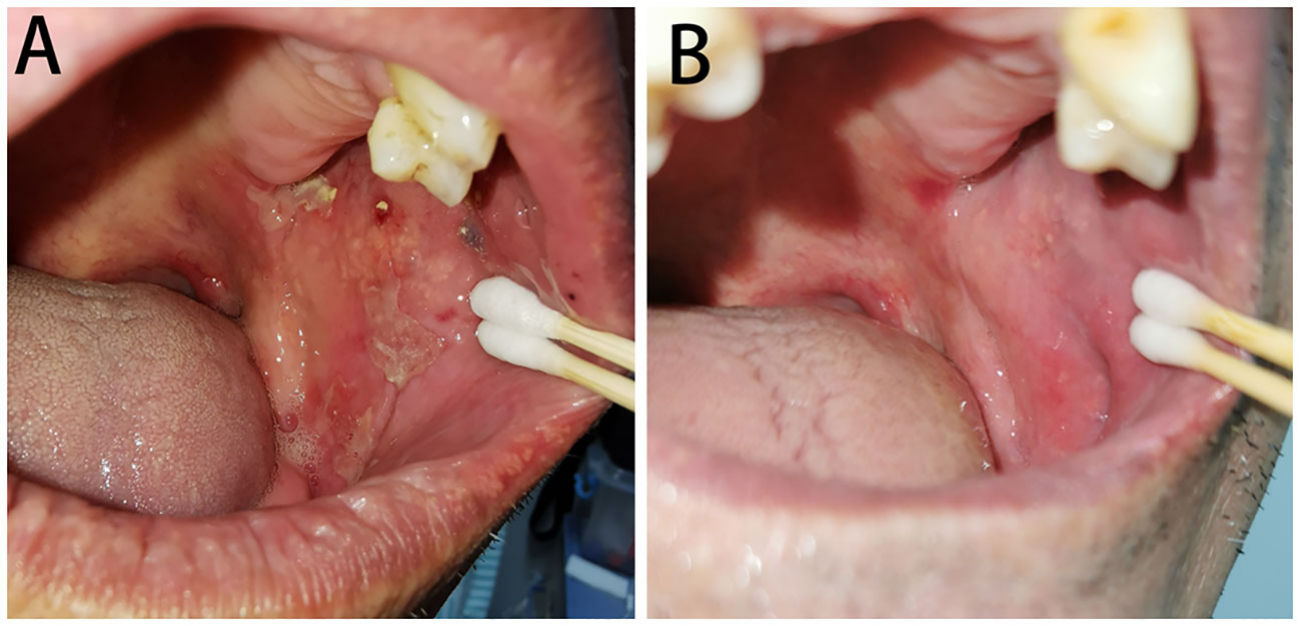
Oral lesions of BP with HSV infected before **(A)** and after **(B)** famciclovir therapy. **(A)** Several erythema and erosions covered with yellowish-white pseudomembranes distributed on the left buccal mucosa and were positive for HSV-1 by PCR. **(B)** The oral lesions almost completely healed after a 2-week treatment of famciclovir.

A total of 14 (73.7%) BP patients in the HSV-positive subgroup were added famciclovir (0.25g, three times a day for 2 weeks) with the dosage of glucocorticoid decreased or no change of the existing treatments. After 2 weeks of antiviral therapy, oral mucosa BPDAI scores significantly decreased [decreased oral mucosa BPDAI score, mean (SD), 3.9 (3.1), p<0.001]; 12 (85.7%) patients stopped developing new oral lesions, and eight (57.1%) patients got completely healed for oral lesions (**Figure 3**).

The other five (26.3%) BP patients in the HSV-positive subgroup were not added famciclovir because three were lost to follow-up and two refused to add famciclovir, since they could hardly feel discomfort with their oral lesions. For the two patients who refused to take oral famciclovir, although their existing oral lesions could heal within 1–2 weeks, new lesions continued to appear consistently, when followed up by phone 2 weeks later.

Six patients were retested for HSV on oral lesions after 1 week of famciclovir treatment, and the results showed four (66.7%) were still positive for HSV; another four patients were retested for HSV on oral lesions after 2 weeks of famciclovir treatment, and all patients were detected negative for HSV.

## 4 Discussion

Although several studies have investigated the prevalence or clinical features of HSV infection in autoimmune bullous disease, they mainly focused on pemphigus vulgaris (PV). PV is the most common intraepidermal bullous dermatosis, which mainly manifests as erosions on the oral mucosa and can be accompanied by flaccid blisters and erosions on the skin sometimes (18). Konda et al. found that 23 out of 60 (38.3%) PV patients were positive for HSV, and male sex, the presence of fissures, hemorrhagic crusts, erosions with angulated margins, linear erosions, and raised erythrocyte sedimentation rate were significantly associated with HSV infection (19). However, they analyzed the clinical features of HSV infection in oral and skin lesions of PV simultaneously. The markedly different manifestations between oral and skin lesions of PV might have some influence on the results. Considering the significantly different characteristics between skin and oral lesions of PV and the difficulty of identifying HSV infection in oral lesions of PV, we carried out a prospective cross-sectional study targeted only oral lesions of PV and found that 17 out of 40 (42.5%) PV patients were positive for HSV in oral lesions and PV patients with HSV infection on oral lesions were more likely to have oral lesions located in the labial mucosa/posterior aspect of the pharynx, covered with pseudomembranes/ruptured blister walls, pain, in the active period, and with a higher pemphigus vulgaris area index score of oral lesions (20). In addition, Baum et al. found that PV patients with HSV-positive oral lesions had a higher C-reactive protein level, Pemphigus Disease Activity Index score, and shorter time to relapse (21). However, when it comes to the prevalence and clinical markers of HSV infection in BP patients, there was only one study that found that the positive rate of HSV in skin biopsies of BP patients was 10% (2/20) (12). No study had ever explored the prevalence or clinical markers of HSV infection in oral lesions of BP, which may be due to that oral lesions are more common in PV patients than BP patients.

As the first study that explored the prevalence and clinical markers of HSV infection in oral lesions of BP patients, first, we found that nearly half (45.2%) of the patients were positive for HSV in oral lesions, which suggested that HSV infection was common in BP oral lesions and should further arouse the attention that we paid on HSV infection among oral lesions of BP. For this part of the work, we used PCR to detect HSV, which had both a high specificity and sensitivity for HSV detection but could not rule out the possibility of asymptomatic shedding of HSV. To figure out this problem, we enrolled 41 healthy individuals and 32 BP patients without oral lesions as control groups. In the healthy control group, we found that there were 9.8% of healthy individuals detected positive for HSV without any signs or history of HSV infection. In our previous study, the rate of HSV asymptomatic shedding in oral mucosa of healthy individuals was 4.8% (20), which was similar to the 4.7% reported by Tateishi et al. (22) and a little lower than the rate in this study. There were studies showing that the prevalence of HSV infection and asymptomatic shedding increased with age (9, 22). The relatively older age of healthy individuals in our study may explain this difference. In the non-lesion group, none of the BP patients without oral lesions was detected positive for HSV. It was worth noticing that there were two special BP patients who did not have oral lesions at the first time, so we initially enrolled them into the non-lesion group and found that both patients were detected positive for HSV. Interestingly, they developed oral lesions 3 and 5 days later separately, so thereafter, we moved them into the lesion group and retested the HSV on oral lesions, which were still positive. These results suggested that the asymptomatic shedding of HSV in oral mucosa indeed existed in healthy individuals but was rare in BP patients. The usage of immunosuppressants and the ruptured barrier of oral mucosa in BP may play a part in this result.

Second, we found that BP patients in the HSV-positive subgroup were more likely to have inconsistent activity between oral and skin lesions, absence of blister/blood blister in oral lesions, pain for oral lesions, higher dosage of glucocorticoid, and higher 2-week AGC dosage compared with those in the HSV-negative subgroup, and the prediction probability of the combined features for HSV infection in oral lesions of BP was 0.898, which suggested that the parameters could serve as good clinical markers of HSV infection in oral lesions of BP and were helpful in identifying the HSV infection in BP oral lesions in time. There used to be two case reports showing that the HSV infection was likely to be related to the exacerbation of BP (23, 24). The characteristic of inconsistent activity between oral and skin lesions in the HSV-positive subgroup suggested that the influence of oral HSV infection on BP might be topical. It only induced activeness or treatment refractoriness of oral lesions instead of skin lesions. The similar titer of BP180 autoantibody between HSV-positive and HSV-negative subgroups further verified this conclusion. Numerous studies indicate that glucocorticoids can suppress both the innate and adaptive immunity of people (25), which makes glucocorticoids one of the risks of making HSV activated (26–28). There were also some studies showing that glucocorticoids can directly induce HSV reactivation by stimulating the glucocorticoid receptor (11, 29, 30). These can explain the higher dosage of glucocorticoid and higher 2-week AGC dosage in the HSV-positive subgroup.

Third, we observed that the oral mucosa BPDAI scores significantly decreased, and oral lesions of most HSV-positive patients got inactive or even completely healed after famciclovir treatment without the dosage of glucocorticoid increased, which suggested that the early antiviral treatment was good for the control of BP oral lesions and further emphasized the importance of identifying HSV infection in BP oral lesions and adding the anti-viral treatment in time.

Finally, on the one hand, we noticed that four of the six patients were still positive for HSV after 1 week of famciclovir treatment, and all four patients became negative for HSV after 2 weeks of famciclovir treatment. On the other hand, most of the BP patients are in an immunosuppression status with the long-time use of glucocorticoid or other immunosuppressants. Thus, compared with 1 week of antiviral therapy for recurrent HSV infection, 2 weeks of antiviral therapy is more recommended for HSV infection in oral lesions of BP.

There were also several limitations in our study. First, partial laboratory tests were lacking in the lesion group (**Table 4**), which might have some influence on the results. Second, the sample size was not so big, and a multicenter study with a larger sample size is needed to be done to further confirm these results. Finally, although we have set up control groups to show that the asymptomatic shedding of HSV in BP oral mucosa was rare, the direct evidence of active HSV infection, which includes the histological pathology result or viral culture, was absent.

To conclude, HSV infection is common in oral lesions of BP. The inconsistent activity between oral and skin lesions, absence of blister/blood blister in oral lesions, pain for oral lesions, higher currently used glucocorticoid dosage, and higher 2-week AGC dosage in BP patients should alert the physicians to the possibility of HSV infection in oral lesions and prescribe them with 2-week famciclovir in time.

## Data availability statement

The raw data supporting the conclusions of this article will be made available by the authors, without undue reservation.

## Ethics statement

The studies involving humans were approved by Ethics Committee of the Chinese Academy of Medical Sciences, Hospital for Skin Diseases (2021-KY-060). The studies were conducted in accordance with the local legislation and institutional requirements. The participants provided their written informed consent to participate in this study. Written informed consent was obtained from the individual(s) for the publication of any potentially identifiable images or data included in this article.

## Author contributions

HZ: Writing – review & editing, Writing – original draft, Software, Resources, Methodology, Formal analysis, Data curation. MY: Writing – review & editing, Methodology, Formal analysis, Data curation. GL: Writing – review & editing, Visualization, Resources, Data curation. SL: Writing – review & editing, Supervision, Resources, Methodology, Investigation, Data curation. CZ: Writing – review & editing, Resources, Data curation. KJ: Writing – review & editing, Resources, Data curation. SF: Writing – review & editing, Supervision, Resources, Investigation, Funding acquisition, Data curation, Conceptualization.
